# Fault Detection and Identification in the Doubled Attitude and Heading Reference System (AHRS)

**DOI:** 10.3390/s25051603

**Published:** 2025-03-05

**Authors:** Grzegorz Kopecki, Bogusław Dołęga, Paweł Rzucidło

**Affiliations:** Department of Avionics and Control, Faculty of Mechanical Engineering and Aeronautics, Rzeszów University of Technology, al. Powstańców Warszawy 8, 35-959 Rzeszów, Poland; dolbog@prz.edu.pl (B.D.); pawelrz@prz.edu.pl (P.R.)

**Keywords:** AHRS, diagnostic system, redundancy, analytical redundancy, low-cost control systems

## Abstract

This article presents a diagnostic system for two redundant AHRS units. The proposed system enables fault detection and identification, facilitating the design of more efficient flight control systems, particularly in low-cost applications. First, the principles of AHRS operation are first introduced, followed by a detailed description of the diagnostic system and the demonstration of the system’s properties. Simulation results presented in this article confirm the system’s effectiveness in fault detection and identification. The proposed solution can be applied in aeronautical control systems, particularly in UAV applications.

## 1. Introduction

The first dissertations by Beard, Jones, and Willsky on fault detection and identification (FDI) in control systems were published in the early 1970s [1,2,3]. Since then, numerous approaches have been developed in this field. For comprehensive reviews, see works by Gertler, Isermann, Edwards, and Blanke, as well as papers by Mehra, Simani, Frank, Patton, and Zhang [4,5,6,7,8,9,10,11,12]. In the context of flying vehicles, abnormal behavior is often complex and results from multiple causal and co-occurring factors acting simultaneously. The aircraft’s state is measured by a set of sensors providing aerometric and inertial data that characterize its position, speed, and altitude. These measurements are acquired through a data acquisition system composed of multiple dedicated redundant units and subsequently processed to calculate consolidated flight parameters. For a comprehensive analysis of redundancy in aircraft systems, see the survey by Marzat [13].

The attitude and heading reference system (AHRS) is one of the most critical onboard units in an aircraft, providing essential information about pitch, roll, and heading angles. Numerous researchers have focused on the challenges of attitude estimation. Classical principles are well established and thoroughly described, for example, in [14]. Research also addresses correction methods, where Kalman filtering or complementary filtering techniques are commonly applied [15,16,17].

Aviation requires an exceptionally high level of reliability [18]. Ensuring fail-safe operation is a critical task across the entire aviation sector, including UAV systems [19].

To achieve the required level of reliability, the most essential onboard systems are duplicated or triplicated. In the event of a failure in one of these critical systems, redundancy mechanisms are employed. A fault is considered detected when the system identifies the presence of a malfunction. Fault identification occurs when the system determines which specific unit or component is faulty. Failures in measurement systems, where no data are transmitted, are relatively easy to detect and identify. However, certain faults may arise where the system continues to operate, but the output signals are erroneous [20,21]. Fault detection requires comparing two independent measurements of the same physical quantity, while fault identification necessitates the use of tripled measurement systems [22].

In several aircraft systems, implementing tripled sensors poses significant challenges. First, sensor multiplication increases costs, which is particularly problematic for low-cost systems, such as those used in General Aviation and small UAVs [23,24]. Moreover, additional sensors contribute to increased mass, a critical parameter in aviation. Additionally, each added system increases power consumption, which must also be carefully considered. The issue of control and power consumption in measurement and control systems is particularly important in small, electrically powered UAVs. In some solutions, the temperature of angular rate sensors is stabilized by electric heating to enhance performance. However, even if the sensor itself is energy-efficient, the heating system is not. Eliminating a single measurement unit can reduce the overall energy consumption in the system.

To address these challenges, some researchers focus on the application of software redundancy in aviation measurement systems [25,26]. Analytical redundancy enables an extended diagnostic process by leveraging alternative mathematical models. Common approaches include different forms of physical equations, state observers, and estimators, as well as neural and fuzzy models [21].

This article presents a proposal for a diagnostic system based on two AHRS units and analytical methods. It is a novel proposal for aviation, where for fault identification, a tripled AHRS is typically used. The system enables the detection and identification of faulty components within the doubled AHRS. A fault is detected if the system finds information that one of the sensors measuring an X-value is faulty, and it is not known in which AHRS. A fault is identified if the system finds information in which AHRS, a sensor measuring the X-value, is faulty. Fault detection usually takes place before fault identification. The proposed method is applicable to UAV systems, as well as AHRSs used in General Aviation (GA) aircrafts. The implemented system is based on the principles of AHRS operation, which are outlined in the following chapter. Redundant angular rates data are obtained from the attitude and heading angles sent by AHRS. The proposed diagnostic system is then described in detail. The system was analyzed through simulations, and selected results are presented and discussed. Finally, conclusions are provided.

## 2. List of Symbols and the Most Important Definitions

The following symbols are used in this article:

- *P*, *Q*, and *R*—angular rates measured in the aircraft body frame, measured, respectively, around the aircraft *x*, *y*, and *z* axes;
- *a_x_*, *a_y_*, and *a_z_*—accelerations measured, respectively, along the aircraft *x*, *y*, and *z* axes;
- *H_x_*, *H_y_*, *H_z_*—magnetic field strength vector components, measured, respectively, along the aircraft *x*, *y*, and *z* axes;
- *Φ*—aircraft roll angle;
- *Θ*—aircraft pitch angle;
- *Ψ*—aircraft heading;
- X_i_—value of X signal (*P*, *Q*, *R*, *Φ*, *Θ*, *Ψ*, *a_x_*, *a_y_*, *a_z_*, and *Ψ_cor_*) measured or calculated by *i*-th AHRS unit;
- *Φ_G_*, *Θ_G_*, *Ψ_G_*—aircraft roll, pitch, and heading calculated from *P*, *Q*, and *R* before correction;
- *X_cor_*—signal *X* (*Φ*, *Θ*, *Ψ*) used for correction, calculated from accelerations measurement or magnetic field strength vector components;
- *r_x_*—residuals based on hardware redundancy, where x: *P*, *Q*, *R*, *a_x_*, *a_y_*, *a_z_*, *Ψ_cor_*, *Φ*, *Θ*, and *Ψ*;
- *r_xi_*—residuals based on hardware and software redundancy, both values for the same AHRS unit, where x: *P*, *Q*, and *R*—analyzed measurements and *i*—the number of analyzed AHRSs;
- *r_xij_*—residuals based on software redundancy, both values for the same AHRS unit, where x: *P*, *Q*, and *R*—analyzed measurements and *i* and *j*—the number of analyzed AHRSs;
- *Tdet*—time of detection of the fault;
- *Tident*—time of identification of the fault;
- AHRS—attitude and heading reference system.

## 3. Principles of AHRS Operation

**Figure 1:**
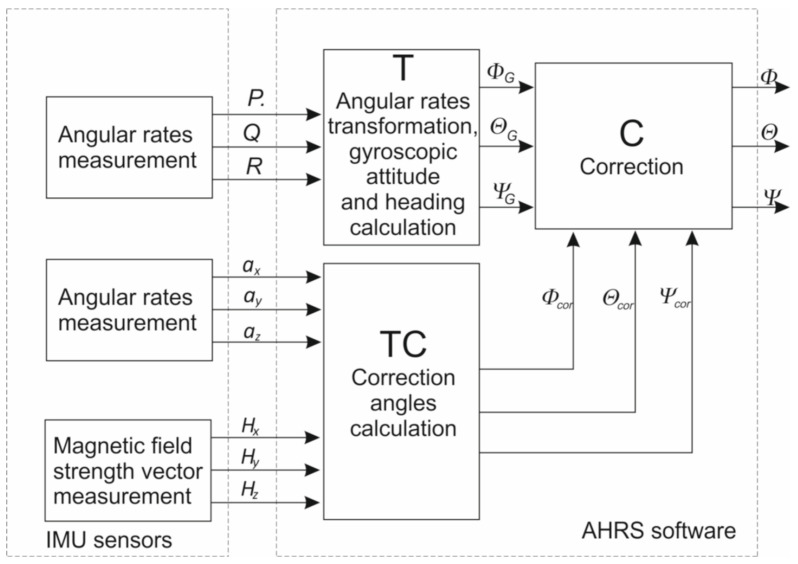
AHRS—general scheme.

Figure 1 presents the general scheme of the AHRS. The AHRS unit sensors include the following: angular rate sensors measuring *P*, *Q*, and *R* in the aircraft body frame (*P*—angular rate around the aircraft *x* axis, *Q*—angular rate around the aircraft *y* axis, and *R*—angular rate around the aircraft *z* axis); acceleration sensors measuring *a_x_*, *a_y_*, and *a_z_* accelerations of the aircraft (*a_x_*—acceleration along the aircraft *x* axis; *a_y_*—acceleration along the aircraft *y* axis; and *a_z_*_—_acceleration along the aircraft *z* axis); and *H_x_*, *H_y_*, and *H_z_* magnetic field strength vector sensors, or a magnetic heading sensor, which measures the components of the magnetic field strength vector or the magnetic heading. The angular rates *P*, *Q*, and *R* are transformed into the Earth frame and then integrated (block T). The transformation typically uses algorithms based on Tait–Bryan angles or quaternion algebra [14,27].

Gyroscopic attitude and heading angles are subject to low-frequency errors, commonly referred to as gyroscopic drift. To minimize the influence of gyroscopic drift, correction is necessary (block C). Typically, Kalman filtering or complementary filtering is used for this correction [14,27]. The Kalman filter minimizes the estimation error variance; however, the estimation process requires significant microcontroller resources. The complementary filter, on the other hand, eliminates sensor frequencies that are prone to error. For gyroscopes, the low-frequency drift is addressed, while for accelerometers and magnetic sensors, high-frequency noise is filtered. The angles used for correction are calculated from the measured accelerations and the magnetic field strength vector (block TC), based on geometric relations.

Attitude and heading angles estimated by the AHRS, along with their derivatives, are functions of several parameters, which can be expressed in the following form:

(1) $$\Phi =f(P,Q,R,\Phi ,\Theta ,\Phi_{cor})$$

(2) $$\Theta =f(P,Q,R,\Phi ,\Theta ,\Theta_{cor})$$

(3) $$\Psi =f(P,Q,R,\Phi ,\Theta ,\Psi_{cor})$$

The complexity of the calculated angles can be used as additional diagnostic information for the system, enabling fault identification through the use of two AHRS units.

## 4. Structure of Diagnostic System

**Figure 2:**
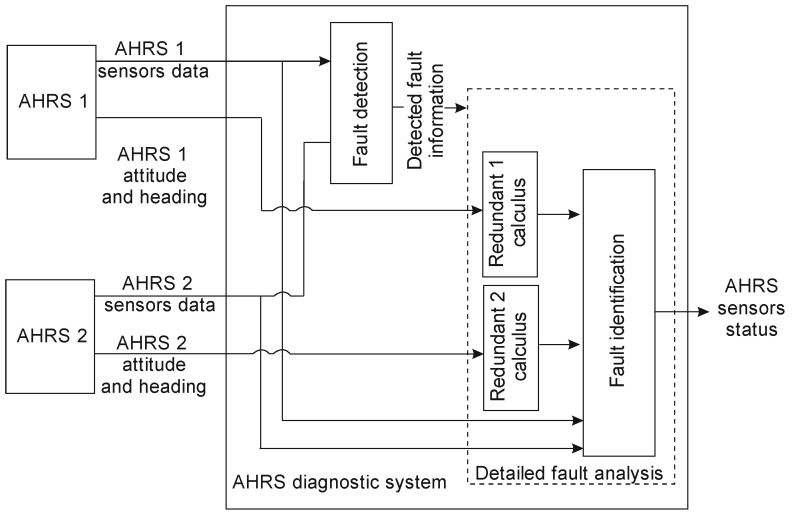
The structure of the diagnostics system.

The designed system consists of two AHRS units. Figure 2 illustrates the concept of the diagnostic system.

Initially, the general AHRS diagnostic system analyzes signals measured directly from the sensors of both AHRS 1 and AHRS 2. This enables the detection of faulty sensors. Fault recognition and identification are based on the analysis of residuals. In this article, a residual is defined as the difference between two measurements or estimates of the same physical quantity. If the system operates correctly (i.e., the residual values remain within acceptable boundaries), no further steps are necessary. This approach enhances system efficiency, which is particularly important in low-cost systems.

After fault detection, the fault identification process is required. For this process, three signals of the same physical quantity are necessary. To replicate the data used for diagnostics, the Redundant Calculus 1 and Redundant Calculus 2 blocks are employed. These blocks calculate angular rates in the body frame, which are derived from the attitude and heading angles, as follows:

(4) $$\left[\begin{array}{c} P_{xR}\\ Q_{xR}\\ R_{xR} \end{array}\right]=\left[\begin{array}{ccc} 1 & 0 & -\sin\Theta_{x}\\ 0 & \cos\Phi_{x} & \cos\Theta_{x}\sin\Phi_{x}\\ 0 & -\sin\Phi_{x} & \cos\Theta_{x}\cos\Phi_{x} \end{array}\right]\left[\begin{array}{c} \dot{\Phi_{x}}\\ \dot{\Theta_{x}}\\ \dot{\Psi_{x}} \end{array}\right]$$

where *x*—the number of AHRS.

Six additional signals significantly enhance the capability of fault identification. Equations (5)–(7) define the residuals of two AHRSs, incorporating both hardware and software redundancy. There are 10 residual values based on hardware redundancy, as well as 12 residual values based on software redundancy (see equations and Table 1)

Residuals based on hardware redundancy are expressed as follows:

(5) $$r_{X}=\left|X_{1}-\Theta X_{2}\right|$$

where X—the analyzed value (*P*, *Q*, *R*, *a**_x_***, *a_y_*, *a_z_*, *Ψ_cor_*, *Φ*, *Θ*, and *Ψ*)

Residuals based on software redundancy are expressed as follows:

(6) $$r_{Xi}=\left|X_{i}-X_{ri}\right|$$

where:

- $r_{Xi}$—the residual value between measurement and estimate from the same AHRS unit;
- X—the analyzed value (*P*, *Q*, and *R)*;
- *i*—the AHRS number.

(7) $$r_{Xij}=\left|X_{i}-X_{rj}\right|$$

where

- $r_{Xij}j$—residual values between the real value from i-th AHRS and the estimate from j-th AHRS.

Hardware redundancy enables only fault detection. The introduction of software redundancy results in a higher number of signals. Importantly, the available number of signals exceeds the minimal number necessary for fault identification. Different residuals exhibit varying sensitivity to different faults. To facilitate the selection of an appropriate set of residuals and support fault detection analysis, a binary diagnostic matrix is designed (Table 1). Each column of the matrix represents a specific sensor fault, while each row corresponds to a residual. A value of 1 in the matrix indicates that the residual is highly sensitive to the given fault. If the residual is sensitive to the fault but the magnitude of the residual is relatively small, it is denoted by ~1 in Table 1. For analysis of residuals sensitivity, a fault was simulated as a constant value added to the measured signal (for gyroscopes: 1 [deg/s]; for accelerations: 10 [m/s^2^]. For hardware redundancy, a highly sensitive residual is when the error appears as a step signal. For software redundancy, a highly sensitive residual is assumed when the mean value of residual, within 10 s after the fault has appeared, assumes a double value of standard deviation of residual value noises without fault. It is worth mentioning that the distinction between highly sensitive and sensitive residual values was introduced solely to facilitate the selection of residues for further analysis in case of residual value redundancy.

The analysis of Table 1 demonstrates the feasibility of fault identification. Moreover, it facilitates the efficient implementation of the diagnostic process and the selection of the necessary residuals. Below, an analysis of each sensor fault is presented. Seven groups of sensors measuring the same physical quantities were identified.

1. Fault in one of the gyroscopes

A fault in the *P* gyroscope is detected through the analysis of *r_P_*. The next step is to identify which specific sensor is faulty. The analysis of Table 1 indicates that examining *r_P1_* and *r_P2_* enables fault identification. To enhance the efficiency of the identification process, the analysis of the integrated absolute values of *r_P1_* and *r_P2_* is considered. Integration begins at the moment of fault detection. The faulty signals are analyzed as follows:

$$\mathrm{If}\;\int_{Tdet}^{Tident}r_{P1}dt\geq n\cdot \int_{Tdet}^{Tident}r_{P2}dt\;\mathrm{then}\;\mathrm{the}\;P_{1}\;\mathrm{signal}\;\mathrm{is}\;\mathrm{considered}\;\mathrm{faulty}.\;\mathrm{If}\;\int_{Tdet}^{Tident}r_{P2}dt\geq n\cdot \int_{Tdet}^{Tident}r_{P1}dt\;\mathrm{then}\;\mathrm{the}\;P_{1}\;\mathrm{signal}\;\mathrm{is}\;\mathrm{considered}\;\mathrm{faulty}.$$

where:

- *n*—the assumed multiplier, n>1. The assumed multiplier value was chosen experimentally. If *n* is too small, fault identification can be wrong. If *n* is too large, the identification time is significant.
- Tdet—detection time.
- Tident—identification time.

If none of the above conditions are met, the error remains unidentified.

Similarly, errors in the ***Q*** and ***R*** gyroscopes are analyzed in the event of fault detection. For the ***Q*** gyroscope, the faulty signals are analyzed as follows:

$$\mathrm{If}\;\int_{Tdet}^{Tident}r_{Q1}dt\geq n\cdot \int_{Tdet}^{Tident}r_{Q2}dt,\;\mathrm{then}\;\mathrm{the}\;\mathrm{signal}\;Q_{1}\;\mathrm{is}\;\mathrm{considered}\;\mathrm{faulty}.\;\mathrm{If}\;\int_{Tdet}^{Tident}r_{Q2}dt\geq n\cdot \int_{Tdet}^{Tident}r_{Q1}dt,\;\mathrm{then}\;\mathrm{the}\;\mathrm{signal}\;Q_{2}\;\mathrm{is}\;\mathrm{considered}\;\mathrm{faulty}.$$

For the *R* gyroscope, the faulty signals are analyzed as follows:


$$\mathrm{If}\;\int_{Tdet}^{Tident}r_{R1}dt\geq n\cdot \int_{Tdet}^{Tident}r_{R2}dt,\;\mathrm{then}\;\mathrm{the}\;\mathrm{signal}\;R_{1}\;\mathrm{is}\;\mathrm{considered}\;\mathrm{faulty}.\;\mathrm{If}\;\int_{Tdet}^{Tident}r_{R2}dt\geq n\cdot \int_{Tdet}^{Tident}r_{R1}dt,\;\mathrm{then}\;\mathrm{the}\;\mathrm{signal}\;R_{2}\;\mathrm{is}\;\mathrm{considered}\;\mathrm{faulty}.$$

2. Fault in the *a_x_* or a_y_ accelerometer

As shown in Figure 2, and analogically to the previous case (faulty gyroscope signals), the first step is error detection using hardware redundancy. The fault is detected by analyzing *r_ax_* in the case of an *a_x_* fault or *r_ay_* in the case of an *a_y_* fault. The next step is to identify the faulty sensor. The analysis of Table 1 indicates that the examination of *r_Q1_* and *r_Q2_* enables fault identification. The conditions are analogous to those for the faulty gyroscope analysis. For *a_x_* acceleration faults, the faulty signals are analyzed as follows:

$$\mathrm{If}\;\int_{Tdet}^{Tident}r_{Q1}dt\geq n\cdot \int_{Tdet}^{Tident}r_{Q2}dt,\;\mathrm{then}\;\mathrm{the}\;\mathrm{signal}\;a_{x1}\;\mathrm{is}\;\mathrm{considered}\;\mathrm{faulty}.\;\mathrm{If}\;\int_{Tdet}^{Tident}r_{Q2}dt\geq n\cdot \int_{Tdet}^{Tident}r_{Q1}dt,\;\mathrm{then}\;\mathrm{the}\;\mathrm{signal}\;a_{x2}\;\mathrm{is}\;\mathrm{considered}\;\mathrm{faulty}.$$

For ay acceleration faults, the faulty signals are analyzed as follows:

$$\mathrm{If}\;\int_{Tdet}^{Tident}r_{P1}dt\geq n\cdot \int_{Tdet}^{Tident}r_{P2}dt,\;\mathrm{then}\;\mathrm{the}\;\mathrm{signal}\;a_{y1}\;\mathrm{is}\;\mathrm{considered}\;\mathrm{faulty}.\;\mathrm{If}\;\int_{Tdet}^{Tident}r_{P2}dt\geq n\cdot \int_{Tdet}^{Tident}r_{P1}dt,\;\mathrm{then}\;\mathrm{the}\;\mathrm{signal}\;a_{y2}\;\mathrm{is}\;\mathrm{considered}\;\mathrm{faulty}.$$

Fault detection in the acceleration channel is possible only during a semi-straight-line flight. In some cases, where the difference between the correct and faulty signals is not significant, yet a fault is still detected, the identification process may be prolonged. In such instances, an additional analysis of the geometric sum of all accelerations measured by each gyroscope can also be utilized.

3. Fault in the *a_z_* accelerometer

The fault is detected through the analysis of *r_az_*. The next step is to identify which sensor is faulty. The analysis of Table 1 indicates that identifying the fault using analytical redundancy is inefficient. In AHRSs, the acceleration *a_z_* is used to measure correction angles. Correction is applied only during a non-accelerated flight. To improve the efficiency of the diagnostic system, an analysis of the total acceleration of each AHRS during non-accelerated flight is performed. If the geometric sum of the acceleration vector components deviates from the expected gravity vector, the acceleration measurement is considered faulty. A similar approach can be applied when analyzing faults in the a_x_ and a_y_ signals.

4. Fault in one of the magnetic heading measurements

The fault is detected through the analysis of *r_Ψcor_*. Unfortunately, the analysis of Table 1 indicates that fault identification using analytical redundancy was inefficient in several cases. In the case of the presented fault, it is necessary to reconfigure the control algorithms and exclude the heading from the navigation process. Despite its disadvantages, such a solution is feasible in certain scenarios.

The presented description demonstrates the possibility of fault detection and identification in the most typical cases. Importantly, faults in the pitch and roll channels can be identified, and their proper operation is crucial for safety.

## 5. Simulation Analysis

**Figure 3:**
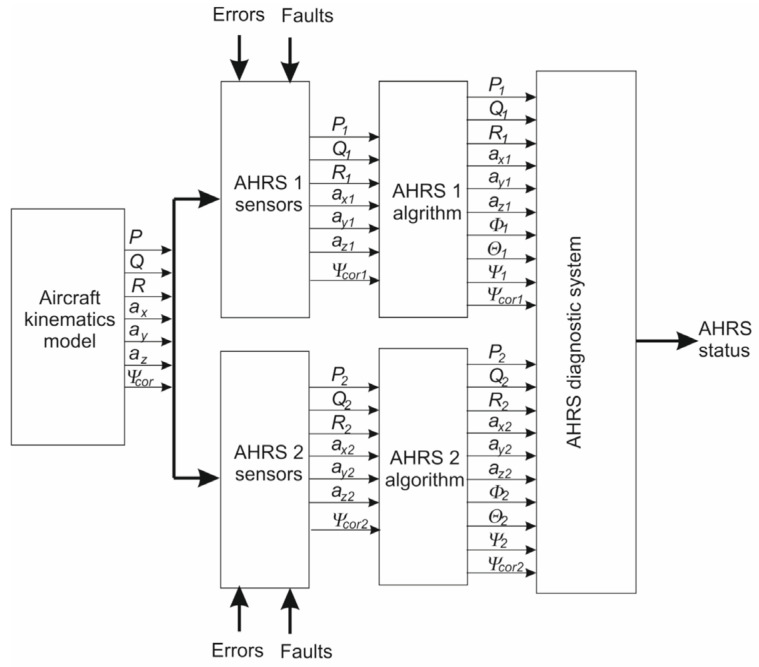
The general scheme of the simulation system.

To illustrate the system’s behavior, a series of simulations was performed. Figure 3 presents the general scheme of the simulation system.

First, the aircraft kinematics model block generates pre-programmed, known aircraft maneuvers, with all turns being coordinated. It sends the angular rates vector (*P*, *Q*, and *R*), the acceleration vector (*a_x_*, *a_y_*, and *a_z_*), and the corrected heading. Flight parameters are transmitted to the AHRS 1 and AHRS 2 sensor blocks, where typical errors (such as noise and low-frequency errors) are introduced, along with simulated faults.

The measured signals are then sent to the AHRS algorithm blocks, which provide information about attitude and heading. AHRS measurements, along with the calculated attitude and heading, are transmitted to the AHRS diagnostic system, whose structure is presented in Figure 2. The diagnostic system provides information on the current AHRS status.

**Figure 4:**
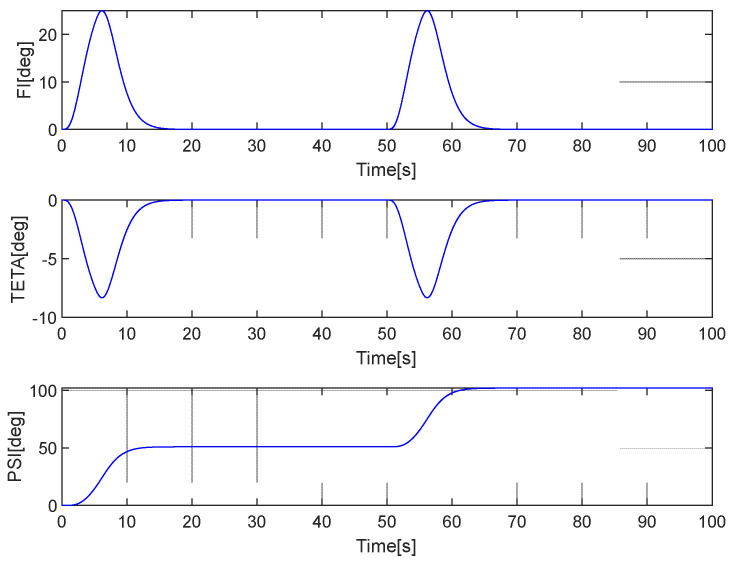
Simulated maneuvers.

Figure 4 presents the simulated maneuvers.

To simulate the performance of real sensors, white noise and constant errors were introduced. Simulated sensor errors are based on previous works [27,28]. They are typical for different classes of FOG gyroscopes.

Modeled sensor errors are as follows:

AHRS 1 errors:

It was assumed that the AHRS 1 sensors were of very good quality.

Acceleration measurements (*a_x_*, *a_y_*, and *a_z_*, respectively):

Mean error values: 0.22073 [m/s^2^], −0.0055 [m/s^2^], and 0.1962 [m/s^2^].

Error variance: 0.00060148 [m^2^/s^4^], 0.00075449 [m^2^/s^4^], 0.00055432 [m^2^/s^4^].

Angular rate measurements (*P*, *Q*, and *R* respectively):

Gyroscope biases: 5 [deg/hr], 5.2 [deg/hr], and 6 [deg/hr].

Error variances: 0.0051 [deg^2^/s^2^], 0.0046 [deg^2^/s^2^], and 0.005 [deg^2^/s^2^].

It was assumed that the AHRS 2 unit is of lower quality than the AHRS 1, but it can still provide very accurate measurement information. The sensor error parameters are as follows:

Acceleration measurements (*a_x_*, *a_y_*, and *a_z_*, respectively):

Mean error values: 0.0981 [m/s^2^], 0.14715 [m/s^2^], and 0.007 [m/s^2^].

Error variances: 0.00050909 [m^2^/s^4^], 3.924 e−05 [m^2^/s^4^], and 0.050909 [m^2^/s^4^].

Angular rate measurement (*P*, *Q*, and *R* respectively):

Gyroscope biases: 60 [deg/hr], 40 [deg/hr], and 70 [deg/hr].

Error variances: 0.01 [deg^2^/s^2^], 0.012 [deg^2^/s^2^], and 0.011 [deg^2^/s^2^].

The results of selected fault simulations are shown below. A simulation of a constant error was performed.

- **Case 1: Fault in the *P_1_* gyroscope**

**Figure 5:**
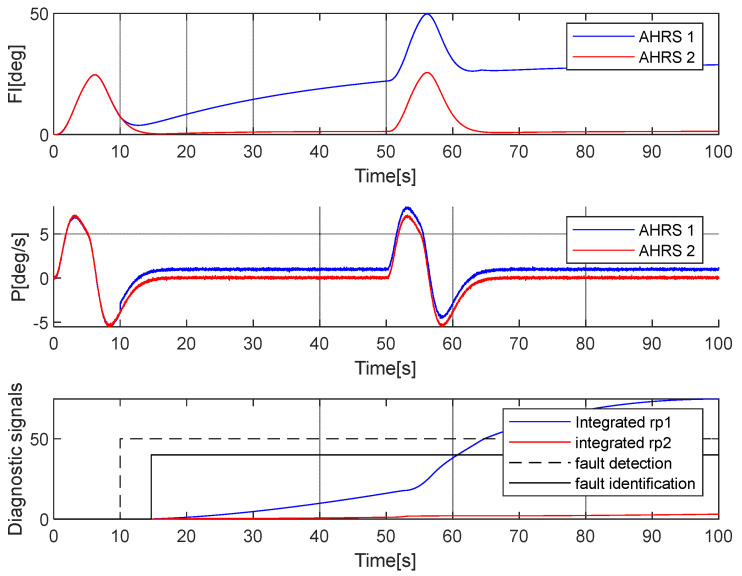
Simulation of faulty measurement of the *P* angular rate in AHRS 1. The fault occurred at the 10th second.

It was assumed that the fault occurred at the 10th second of the flight. The error had a constant value (1°/s), added to the *P_1_* angular speed. Figure 5 presents the simulation results.

As the presented simulations show, the proposed methodology enables efficient fault identification. Similar results were obtained for the *Q* and *R* angular rates, for which an analogous algorithm was implemented. The fault was identified correctly, and the attitude error did not reach dangerous values.

- **Case 2. Fault in the *a_y_* accelerometer**

**Figure 6:**
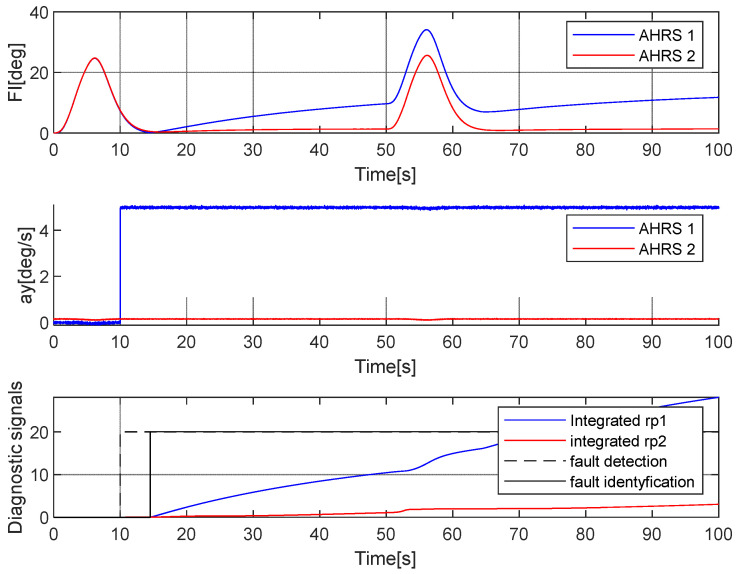
Simulation of the faulty measurement of the *a_y_* acceleration in AHRS 1. The fault occurred at the 10th second.

It was assumed that the fault occurred at the 10th second of the flight. The error was a constant value (10 m/s^2^) added to the ay1 acceleration. Figure 6 presents the simulation results.

Similar to the previous case, the error was detected immediately after its appearance and was identified properly. Similar results were obtained in the case of a faulty *a_x_* gyroscope, for which the algorithm is analogous to the one presented.

- **Case 3. Fault in the *a_z_* accelerometer**

**Figure 7:**
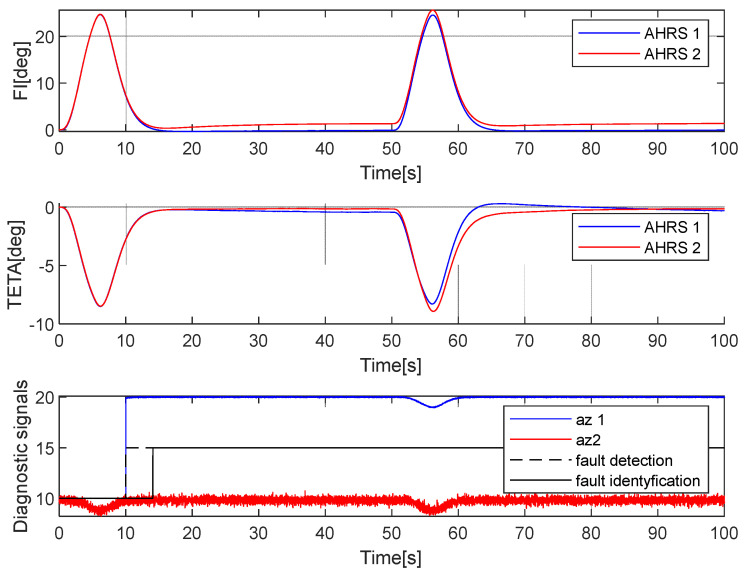
Simulation of the faulty measurement of the *a_z_* acceleration in AHRS 1. The fault appeared at the 10th second.

In an analysis of cases presented earlier in this article, it was assumed that the fault occurred at the 10th second of the flight. The error had a constant value (10°/s) added to the *a_y_* acceleration. Figure 7 presents the simulation results.

First, it has to be mentioned that a serious fault in the *a_z_* accelerometer did not lead to significant errors in the measurement of the attitude angles. The methodology applied for fault detection and identification was effective.

The simulation results are very promising, and faults in angular rate sensors, as well as faults in accelerometers, can be detected and identified using two physical signals. Unfortunately, an error in the measurement of the yaw/heading angle used for correction proved difficult to identify with only two signals. The authors did not obtain satisfactory results for this case.

## 6. Conclusions

The attitude and heading reference system is one of the most important components on board an aircraft. In the case of its faulty operation, the avionics system is significantly endangered. To avoid such dangerous situations, redundant units are used. For fault identification, three signals of the same value are necessary. In the case of a system with duplicated AHRS units, a more advanced diagnostic system is required for fault identification. Such a system was presented in this article. Faulty gyroscope signals were detected and identified correctly. Fault detection was immediate. The time required for fault identification was only a few seconds, and until the fault was identified, it did not significantly affect the measured attitude angles. Proper fault detection does not depend on aircraft maneuvers; for proper fault identification, a correction based on accelerations should be switched on. In AHRSs for aviation, correction is switched on during a straight-line flight. Therefore, for the diagnostic system to function properly (fault identification), a semi-straight-line flight is necessary. This shows that reconfiguration of the control scenario and at least a temporary interruption of the mission scenario are required. For proper fault identification, straight-line flight is needed.

If the fault is identified, the faulty AHRS data should not be taken into consideration; instead, the mission should continue using the properly functioning unit. If two AHRS are built with sensors of different quality (for example, FOG and MEMS gyroscopes), and a single sensor in the better AHRS is faulty, reconfiguration of the measurement system algorithm should be considered, and the properly measured data from the faulty unit should be obtained to achieve better quality of the attitude and heading estimation.

It was pointed out that the identification of the heading correction measurement error was not satisfactory. Fortunately, in the case of a faulty heading measurement, the control system can be reconfigured, and, for example, the GNSS track angle can be used for aircraft control. Reconfiguration of the entire measurement system scenarios, as well as the impact of improper measurements on system behavior, are interesting topics for analysis, and such research is being carried out. A detailed analysis of different faults, their detectability and identification, and their impact on measured signals will also be considered. A topic of high importance for further research is the quantitative analysis of different faults and the possibility of their proper identification. Moreover, data-driven ToMFIR analysis [29,30] appears to be important. The properties of signals measured by faulty sensors is a significant topic, which will show which faults are the most dangerous. For example, the simulation results in this article indicate that, providing the faults in both channels are the same, the faulty *a_z_* measurement is less dangerous than the faulty *a_y_* sensor. In the future, detailed recommendations for system designers will be provided.

## Figures and Tables

**Table 1:** Binary diagnostic matrix of two AHRSs.

	***P*** **_1_**	***Q*** **_1_**	***R*** **_1_**	***ax_1_***	***ay_1_***	***az*** **_1_**	***Ψ*** **_cor1_**	***P*** **_2_**	***Q*** **_2_**	***R*** **_2_**	***ax*** **_2_**	***ay*** **_2_**	***az*** **_2_**	***Ψ*** **_cor2_**
*r_P_*	1							1						
*r_Q_*		1							1					
*r_R_*			1							1				
*r_ax_*				1							1			
*r_ay_*					1							1		
*r_az_*						1							1	
*r_Ψcor_*							1							1
*r_Φ_*	~1				~1	~1		~1				~1	~1	
*r* *_Θ_*		~1		~1					~1		~1		~1	
*r_Ψ_*			~1							~1				~1
*r_P_*_1_	1				1	~1				~1				
*r_Q_*_1_		1		1		~1								
*r_R_*_1_			1				~1							
*r_P_*_2_								1				1	~1	
*r_Q_*_2_									1		1		~1	
*r_R_*_2_														~1
*r_P_*_12_	1							1				1	~1	
*r_Q_*_12_		1							1		1		~1	
*r_R_*_12_			1							~1				
*r_P_*_21_	1				1	~1		1						
*r_Q_*_21_		1		1		~1			1					
*r_R_*_21_			1				~1							~1

## Data Availability

Data are contained within the article.
